# Author Correction: Distinct temporal integration of noradrenaline signaling by astrocytic second messengers during vigilance

**DOI:** 10.1038/s41467-020-17059-x

**Published:** 2020-07-07

**Authors:** Yuki Oe, Xiaowen Wang, Tommaso Patriarchi, Ayumu Konno, Katsuya Ozawa, Kazuko Yahagi, Hirokazu Hirai, Takashi Tsuboi, Tetsuya Kitaguchi, Lin Tian, Thomas J. McHugh, Hajime Hirase

**Affiliations:** 1 RIKEN Center for Brain Science, Wako, Saitama Japan; 2 Center for Translational Neuromedicine, Faculty of Medical and Health Sciences, University of Copenhagen, Copenhagen, Denmark; 3 Department of Biochemistry and Molecular Medicine, University of California, Davis, CA USA; 4 Institute of Pharmacology and Toxicology, University of Zurich, Zurich, Switzerland; 5 Neuroscience Center Zurich, University of Zurich, Zurich, Switzerland; 6 Viral Vector Core, Gunma University Initiative for Advanced Research, Maebashi, Gunma 371-8511 Japan; 7 Department of Neurophysiology & Neural Repair, Gunma University Graduate School of Medicine, Maebashi, Gunma 371-8511 Japan; 8 Department of Life Sciences, Graduate School of Arts and Sciences, The University of Tokyo, 3-8-1 Komaba, Meguro, Tokyo 153-8902 Japan; 9 Laboratory for Chemistry and Life Science, Institute of Innovative Research, Tokyo Institute of Technology, 4259 Nagatsuta-cho, Midori-ku, Yokohama, Kanagawa 226-8503 Japan; 10 Brain and Body System Science Institute, Saitama University, Saitama, Japan

**Keywords:** Cellular neuroscience, Astrocyte

Correction to: *Nature Communications* 10.1038/s41467-020-14378-x, published online 24 January 2020.

The original version of this Article omitted from the author list the 8th author Takashi Tsuboi who is from the ‘Department of Life Sciences, Graduate School of Arts and Sciences, The University of Tokyo, 3-8-1 Komaba, Meguro, Tokyo 153-8902, Japan’ and the 9^th^ author Tetsuya Kitaguchi who is from the ‘Laboratory for Chemistry and Life Science, Institute of Innovative Research, Tokyo Institute of Technology, 4259 Nagatsuta-cho, Midori-ku, Yokohama, Kanagawa 226-8503, Japan’. Consequently, the corrected version of the Acknowledgements removes the following from the original version: ‘Dr. Tetsuya Kitaguchi for providing Pink Flamindo construct.’ Additionally, the following was added to the Author contributions: ‘T.T. and T.K. shared and discussed unpublished Pink Flamindo data.’ This has been corrected in both the PDF and HTML versions of the Article.

