# Supplementary material for: Distinct temporal integration of noradrenaline signaling by astrocytic second messengers during vigilance
Source: Nat Commun. 2020 Jan 24;11:471. doi: 10.1038/s41467-020-14378-x (PMC6981284; doi:10.1038/s41467-020-14378-x)
Supplement: Supplementary file 1 — Supplementary Information [file 41467_2020_14378_MOESM1_ESM.pdf]

Distinct temporal integration of noradrenaline signaling by astrocytic second messengers during vigilance

Oe et al.

Supplementary Information

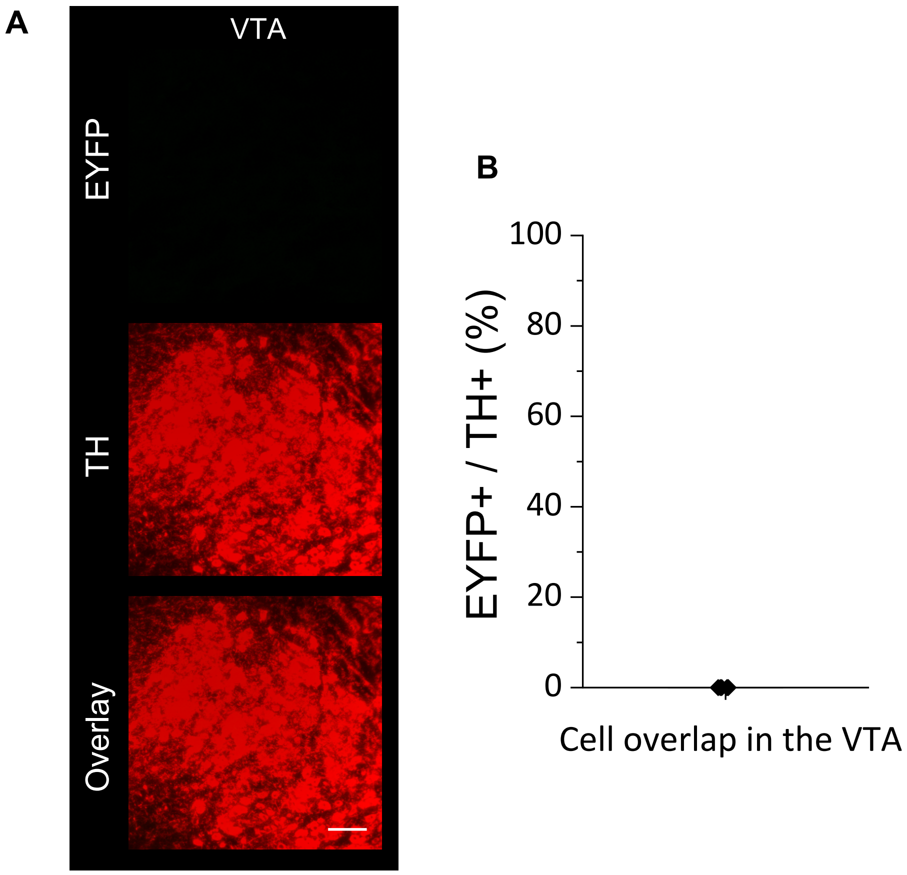

Supplementary Figure 1. No leakage expression in VTA by AAV injection in LC.

VTA of the NAT-Cre mouse with LC injection of AAV-DJ/8-EF1a-DIO-EYFP (thereby expressing EYFP in LC/NA cells) were stained with anti-TH antibody (A). Virtually no cell expressed EYFP in VTA, confirming no leakage of the LC-injected AAV to the VTA (B). Scale bar: 100 μm.

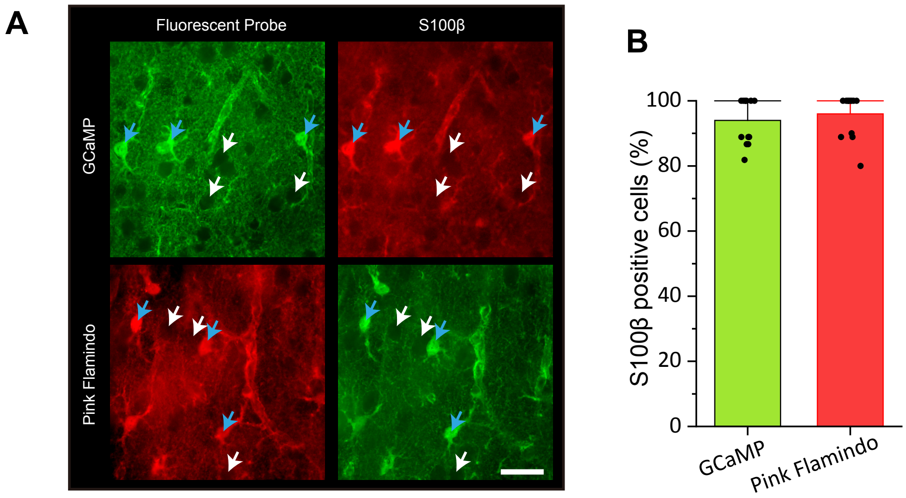

Supplementary Figure 2. Immunohistochemical staining shows that the fluorescent probes are selectively expressed in astrocytes by AAVs with the GFAP promoter.

Cortical tissue expressing GCaMP7 and Pink Flamindo in astrocytes by AAVs with the GFAP promoter was co-stained with S100β (astrocyte marker) immunohistochemistry. Blue arrows indicate fluorescent probe-positive cells that are co-localized with S100β. White arrows are fluorescent probe-negative cells which are not labeled with S100β (A). Nearly all S100β-positive astrocytes expressed fluorescent probes in the AAV-injected area (B) (n = 13 images from 3 mice).

Bar graphs are presented as mean + SEM. Scale bar is 30 μm.

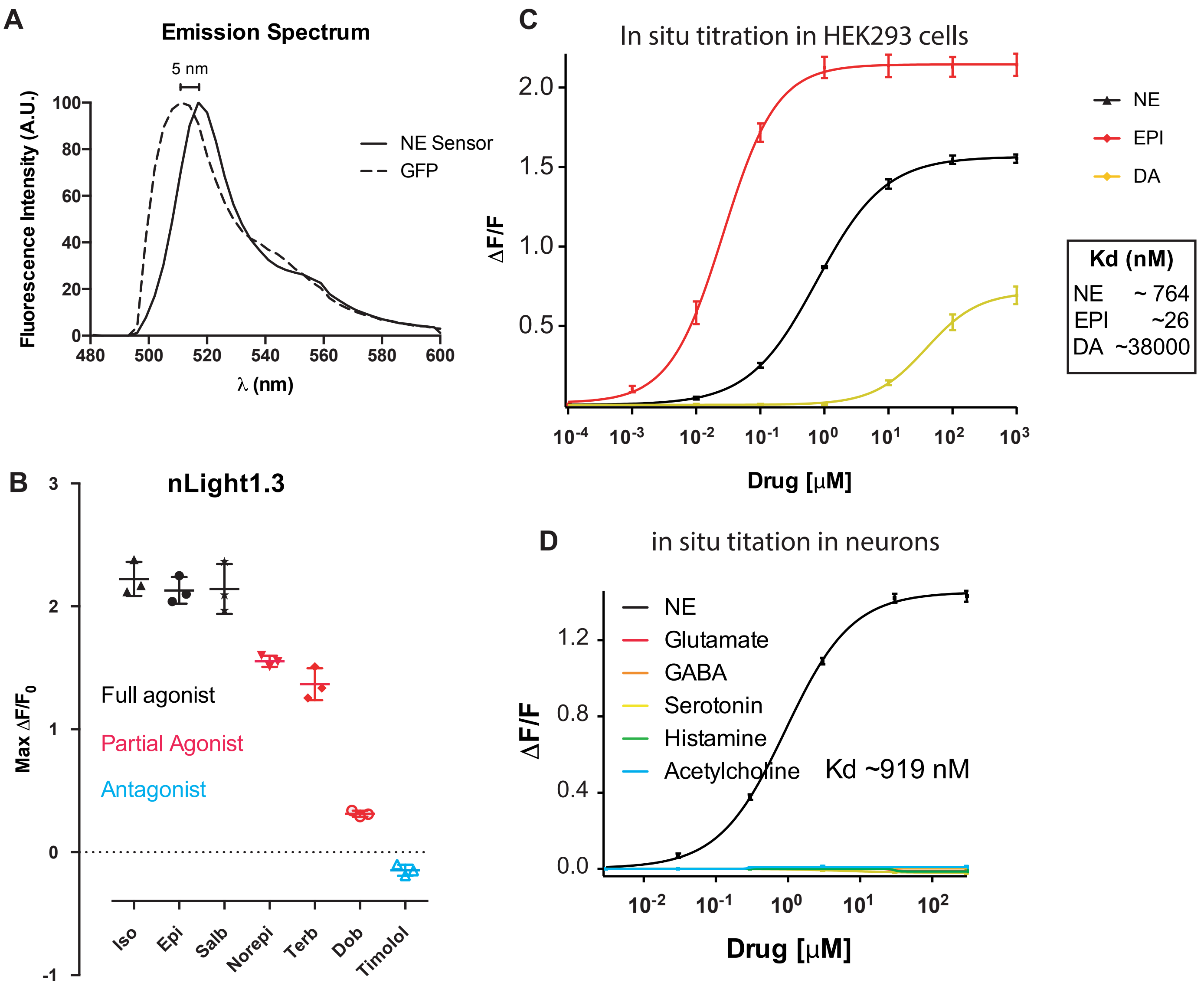

Supplementary Figure 3. by inserting cpGFP into the 3rd intracellular loop of the beta-2 adrenergic receptor (B2AR) followed by screening of ~ 400 linker variants, we identified one with large fluorescent response to NE (ΔF/Fmax = 155 ± 3 %) which is significantly improved compared to the previously reported prototype variants[34] and thus named nLight1.3. HEK 293 cells expression nLight1.3 showed 5nM shift in emission spectra compared to GFP (A). nLight showed various response to pharmacological drugs representing the drug efficacy in activating b2AR receptors (B). In situ titration in HEK 293 cells showed greater response to epi compared to NE; the sensor showed ~50-fold increased selectivity to NE than DA (C). In neurons, the sensor showed negligible response to all the other neurotransmitter tested (D). Experiments in this figure were conducted as previously described[34].

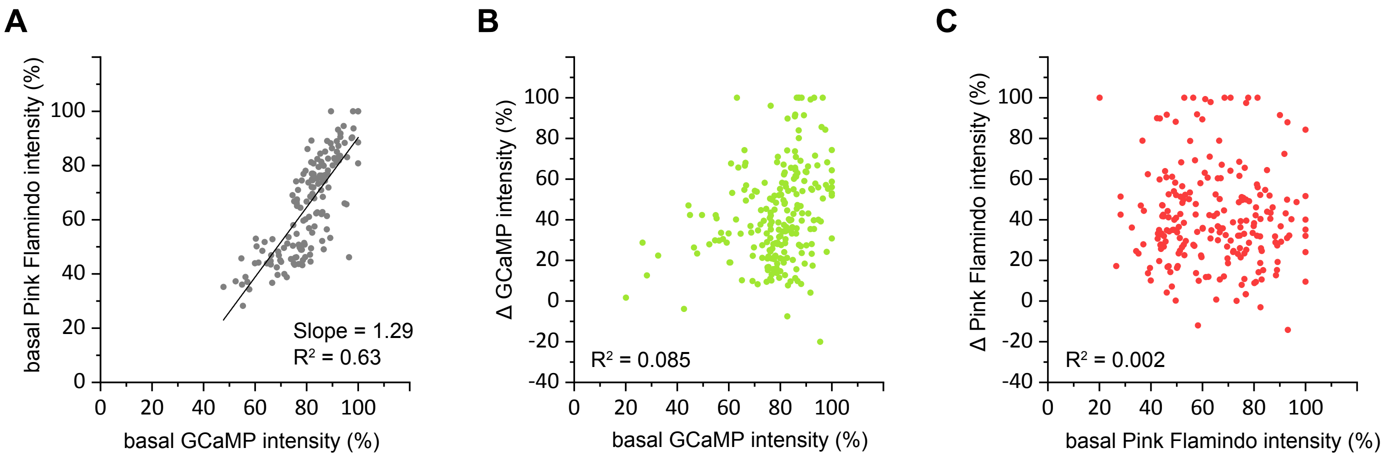

Supplementary Figure 4. Basal fluorescence probe signal does not affect relative fluorescence intensity change.

Basal intensities of GCaMP and Pink Flamindo were measured and plotted from individual astrocytes. The expressions of the two probes are positively correlated among individual cells (A). The basal signal and relative response for each cell is plotted for GCaMP (B) and Pink Flamindo (C). Correlation is not observed in either plots. The low R^2 values suggest that probe expression strength is unlikely the cause of the heterogeneity of response to NA.

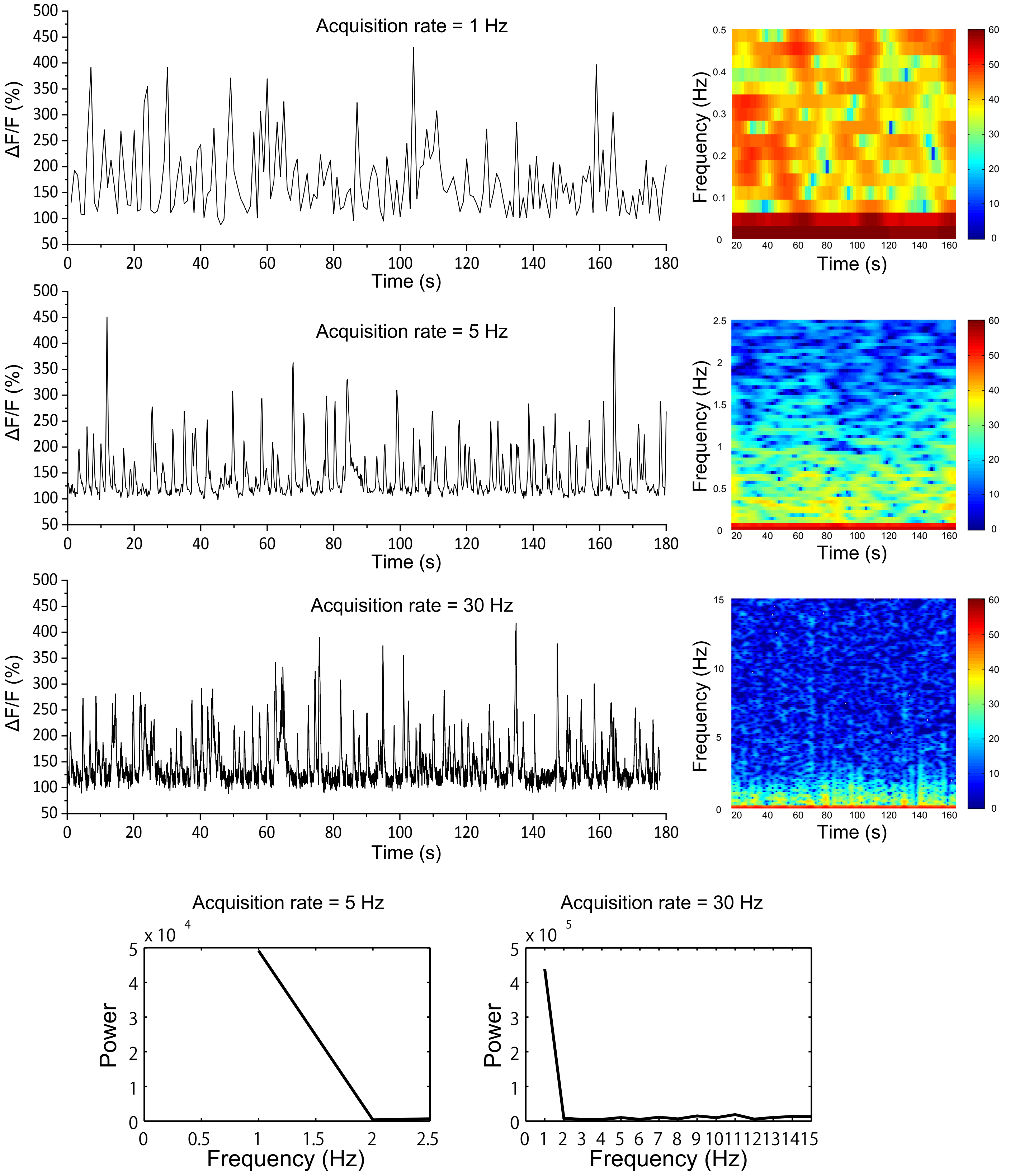

Supplementary Figure 5. 5 Hz acquisition rate is sufficient for imaging GCaMP6.f in cortical NAergic fibers.

GCaMP6.f was expressed in LC NAergic neurons. Images acquired by two-photon microscopy with a resonant scanner show that most of the GCaMP6.f signal signals are captured within 2 Hz sampling. Therefore, 5-Hz and 30-Hz acquisition rates are sufficient for detecting all major Ca^{2+} activities of NAergic fibers.

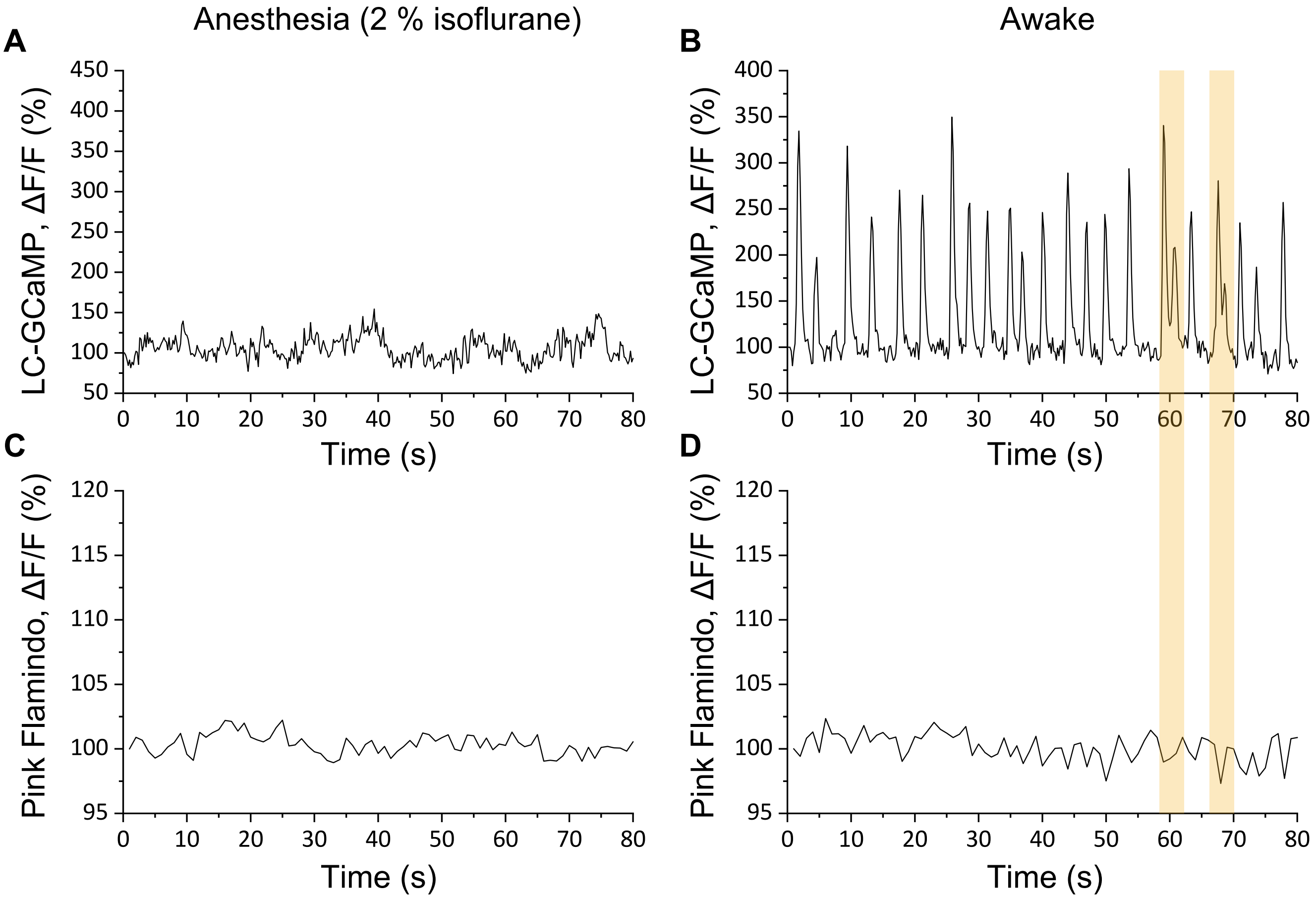

Supplementary Figure 6. Ca^{2+} events in LC/NA axons are dependent on animal states, and infrequent MP signals in arousal states do not accompany cAMP elevation.

LC/NA axons expressing GCaMP6.f were imaged during anesthetized (A) and arousal (B) states in the cortex. Ca^{2+} events were rare in the anesthetized state. In arousal states, infrequent NAergic MP signal occurrences (orange) did not induce Pink Flamindo signal elevations (D). Pink Flamindo signal was stable during the anesthetized state (C).

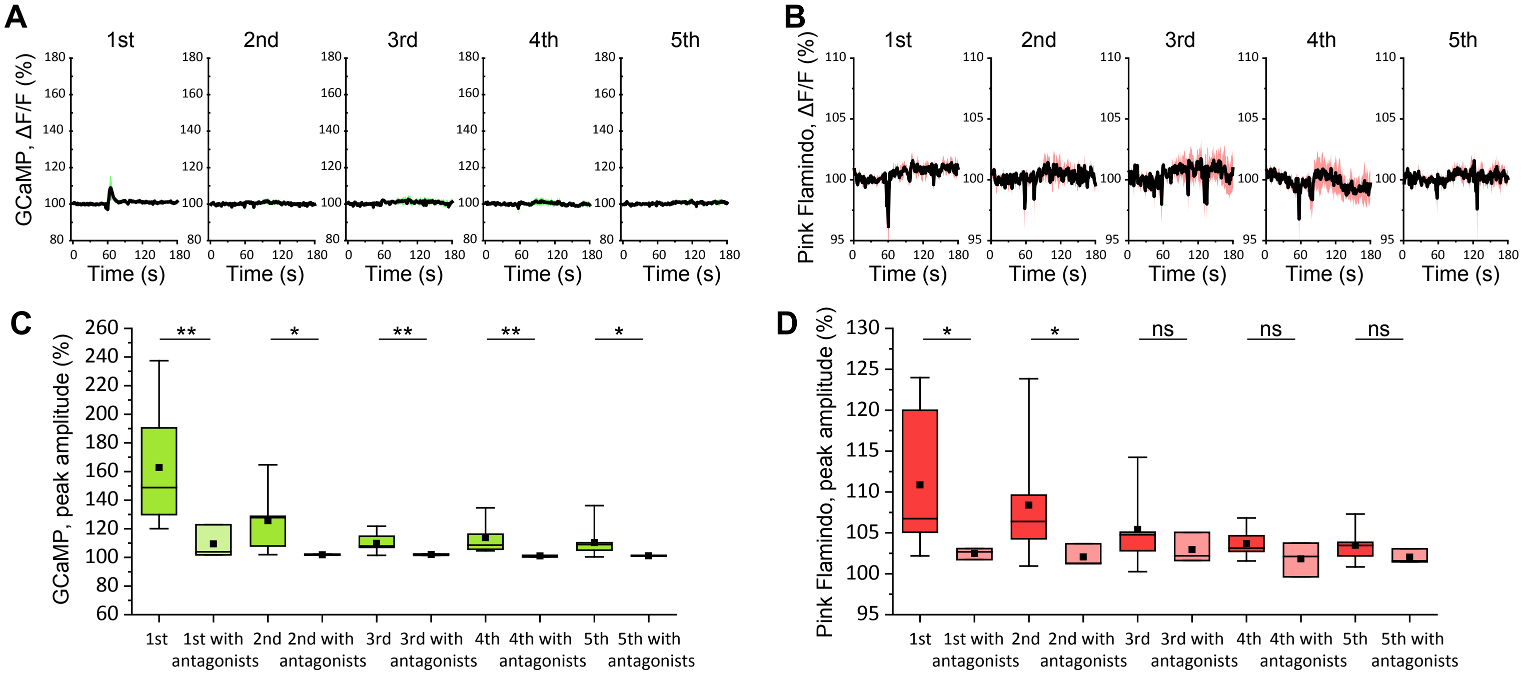

Supplementary Figure 7. Ca^{2+} and cAMP elevations during fear conditioning are induced by NA. Simultaneous Ca^{2+} and cAMP imaging with a cocktail of adrenoceptor antagonists (10 mg/kg prazosin + 10 mg/kg propranolol, i.p. 30 min before imaging) was performed during head-fixed fear conditioning (A, B). The cocktail diminished both Ca^{2+} and cAMP elevations. Peak amplitude was compared with the data from Fig. 6 (C, D). (n = 3 animals, Student' s t-test with Welch' s correction, *p < 0.05, **p < 0.01, ***p < 0.001).

For all box plot figures, middle lines indicate the median, the squares are mean, the boxes show the inter quartile range between the 25th and 75th and upper and lower whisker indicate maximum and minimum values.

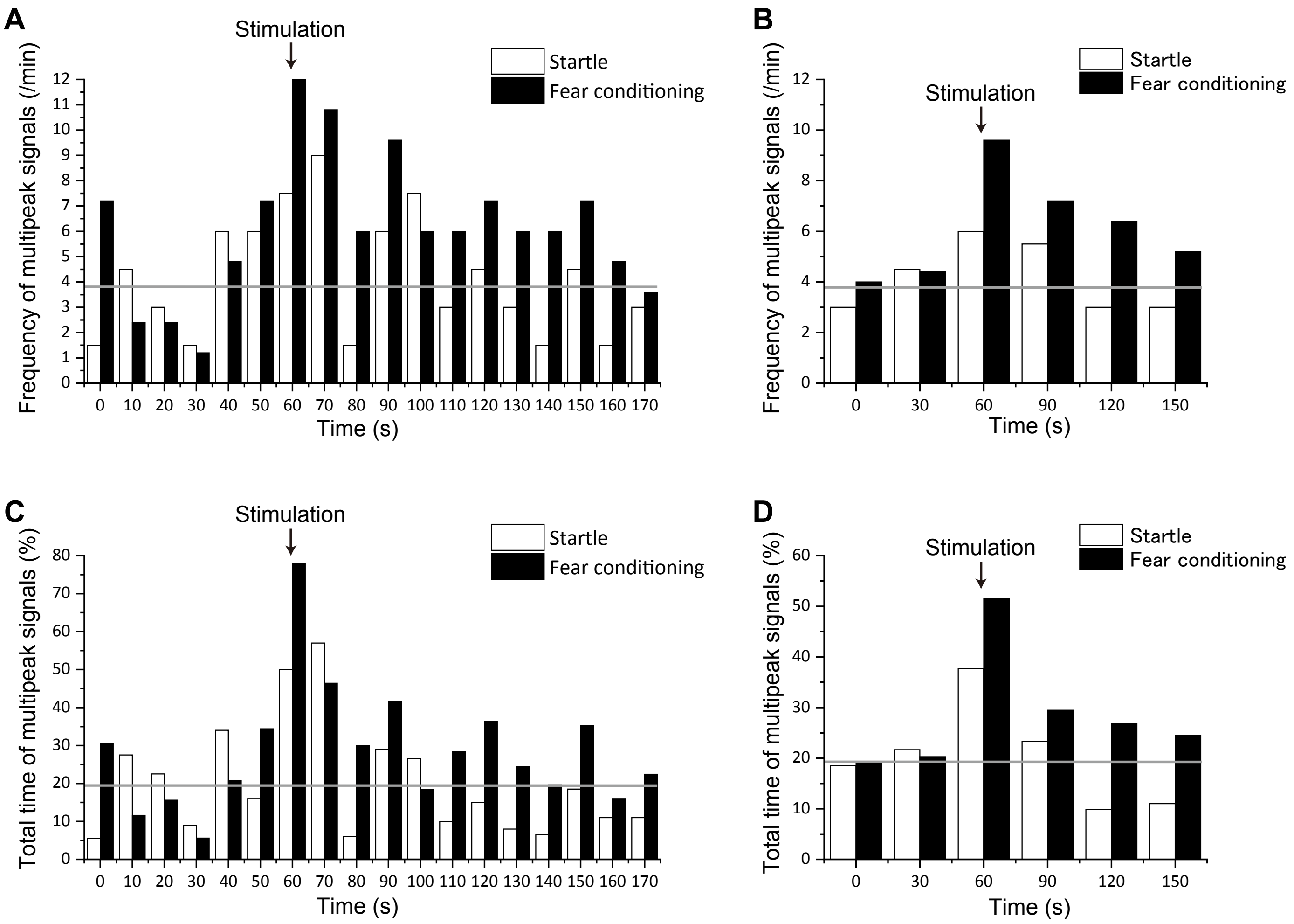

Supplementary Figure 8. Fear conditioning elicits more prolonged NAergic MP signals than startle. GCaMP6.f in LC/NA axons between startle and fear conditioning was compared. Analysis was performed with 10-s (A, C) and 30-s bins (B, D). Both frequency and total time of MP signals showed similar tendencies that NAerigc MP signals last longer after foot shock in fear conditioning than startle. (n = 5 mice for fear conditioning and n = 3 mice for startle)

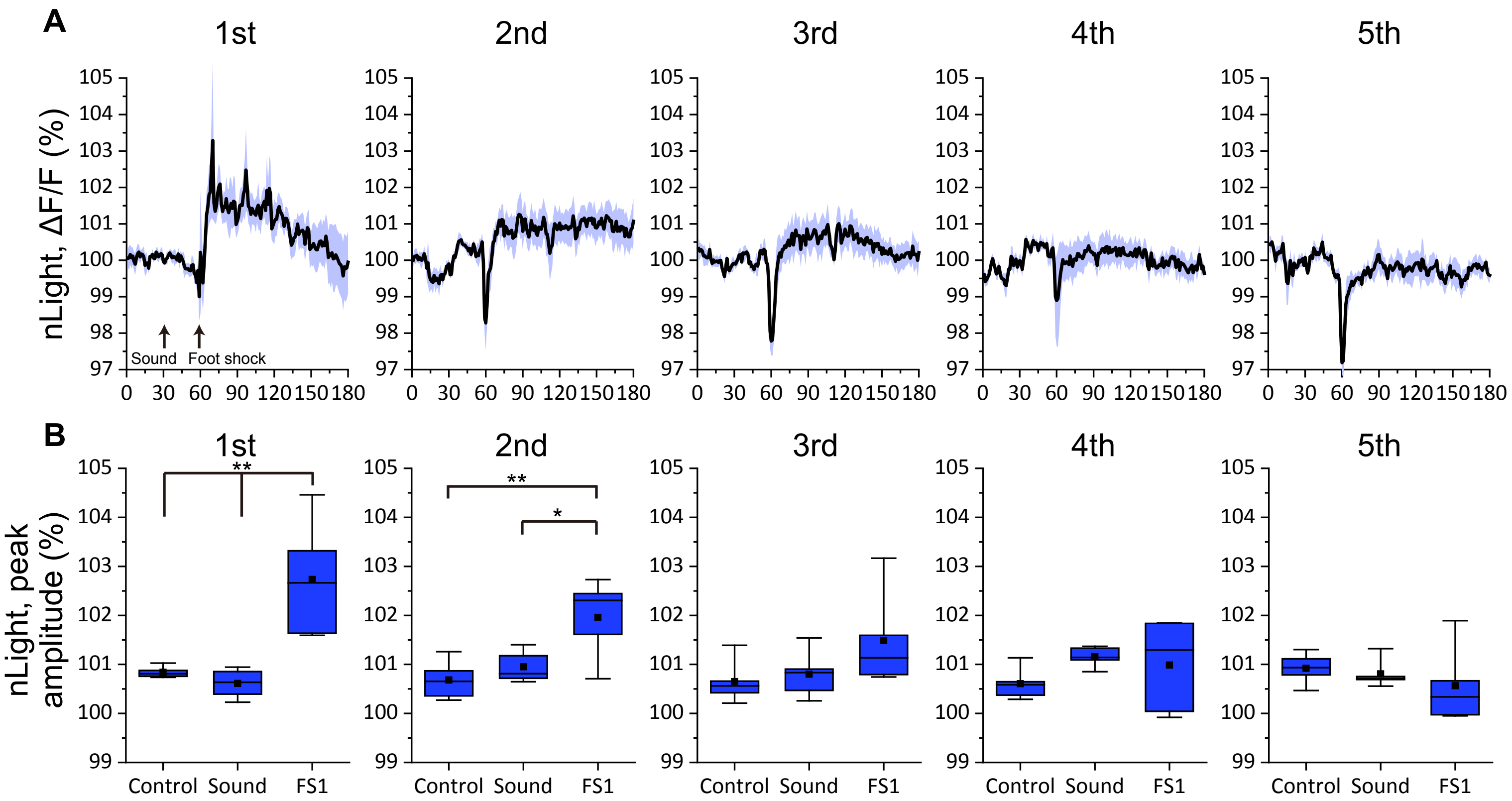

Supplementary Figure 9. Fear conditioning induces extracellular NA elevation in early sessions. nLight expressed in cortical neurons were imaged by two-photon microscopy during fear conditioning. nLight significantly increased after FS in early sessions, but disappeared in later sessions (A, B). These results are consistent with astrocytic second messenger dynamics identified by fear conditioning (Fig. 1), which suggest that Ca^{2+} and cAMP response is governed by extracellular NA level. (n = 5 sessions from 5 mice, one-way ANOVA, p*p < 0.05, **p < 0.01)

For all box plot figures, middle lines indicate the median, the squares are mean, the boxes show the inter quartile range between the 25th and 75th and upper and lower whisker indicate maximum and minimum values.

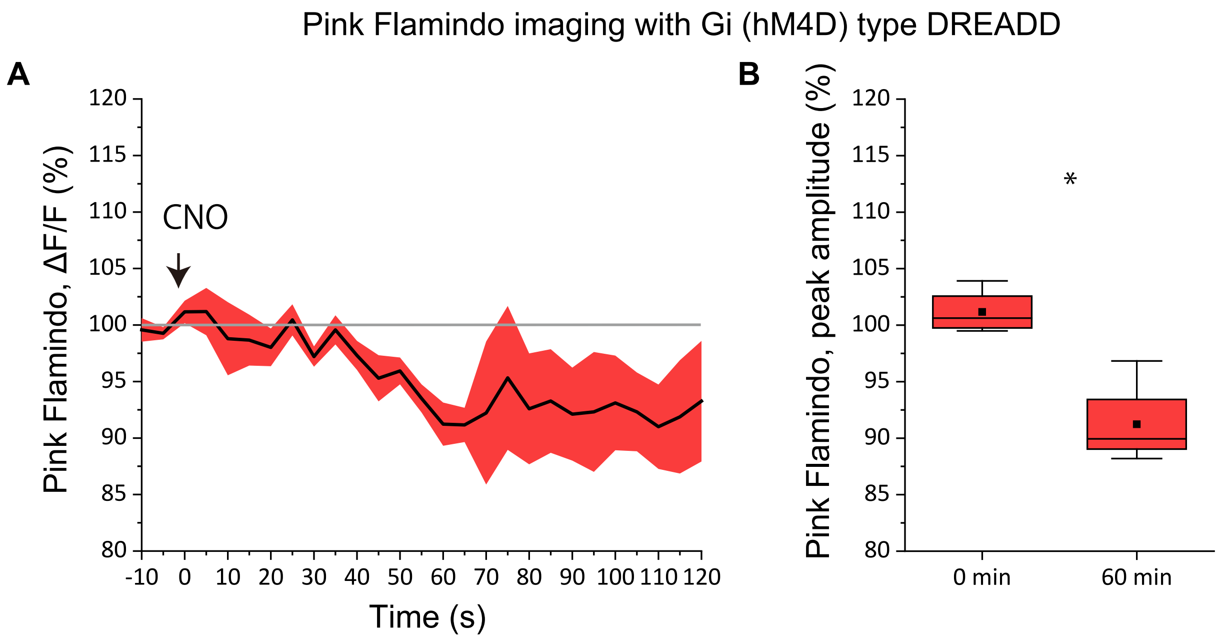

Supplementary Figure 10. Basal cAMP level is measurable by Pink Flamindo and is reduced by Gi signal activation by DREADD.

Gi-type DREADD (hM4D), which reduces cAMP level, was expressed in cortical astrocytes with Pink Flamindo by AAV. Two weeks after injection, Pink Flamindo was imaged with 1040 nm wavelength laser by two-photon microscopy under 1.5 % isoflurane. CNO (1 mg/kg of) was injected 10 min after the start of imaging. cAMP level gradually decreased after CNO injection (A). At 60 min after CNO, the cAMP level was significantly reduced from the basal level (B). (n = 4 sessions from 4 mice, paired t-test, *$p < 0.05$)

For all box plot figures, middle lines indicate the median, the squares are mean, the boxes show the inter quartile range between the 25th and 75th and upper and lower whisker indicate maximum and minimum values.

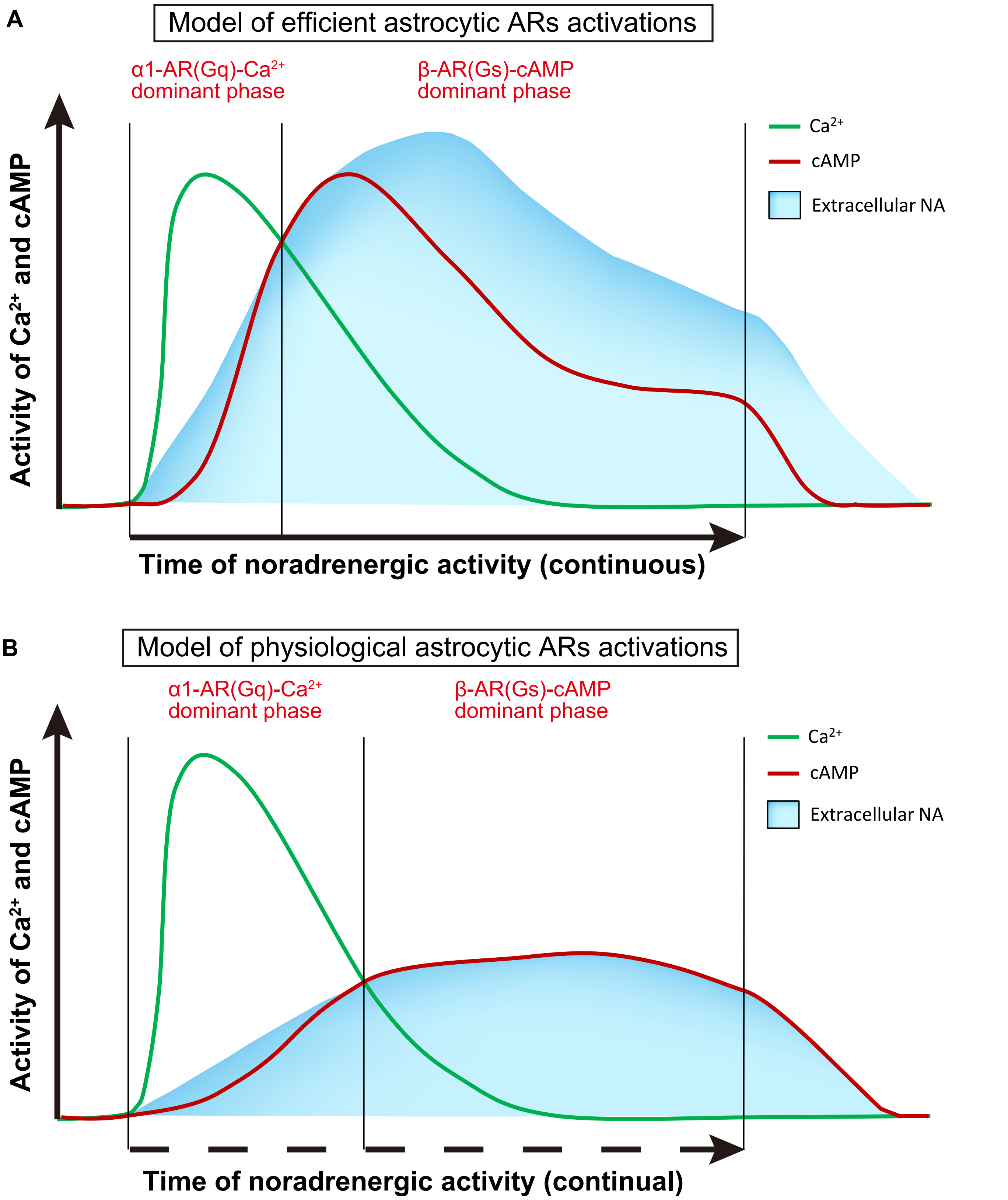

Supplementary Figure 11. A model describing NAergic activity-dependent elevation of the second messengers Ca^{2+} and cAMP in astrocytes.

Efficient elevation of astrocytic second messengers is driven by continuous NAergic activity (A). However, rodent NA neurons do not typically exhibit continuous and intense firing. Instead, NA neurons can discharge in continual phasic patterns. Astrocytic α1-ARs sense the initiation of NAergic activity due to its higher NA affinity and give rise to intracellular Ca^{2+} elevation. Subsequently, the Ca^{2+} level starts to decay regardless of extracellular NA concentration. β-ARs react to extracellular NA with some delay because of its lower NA affinity (B). Astrocytic cAMP elevation accompanies extracellular NA elevation, which is visualized by cellular optical imaging.

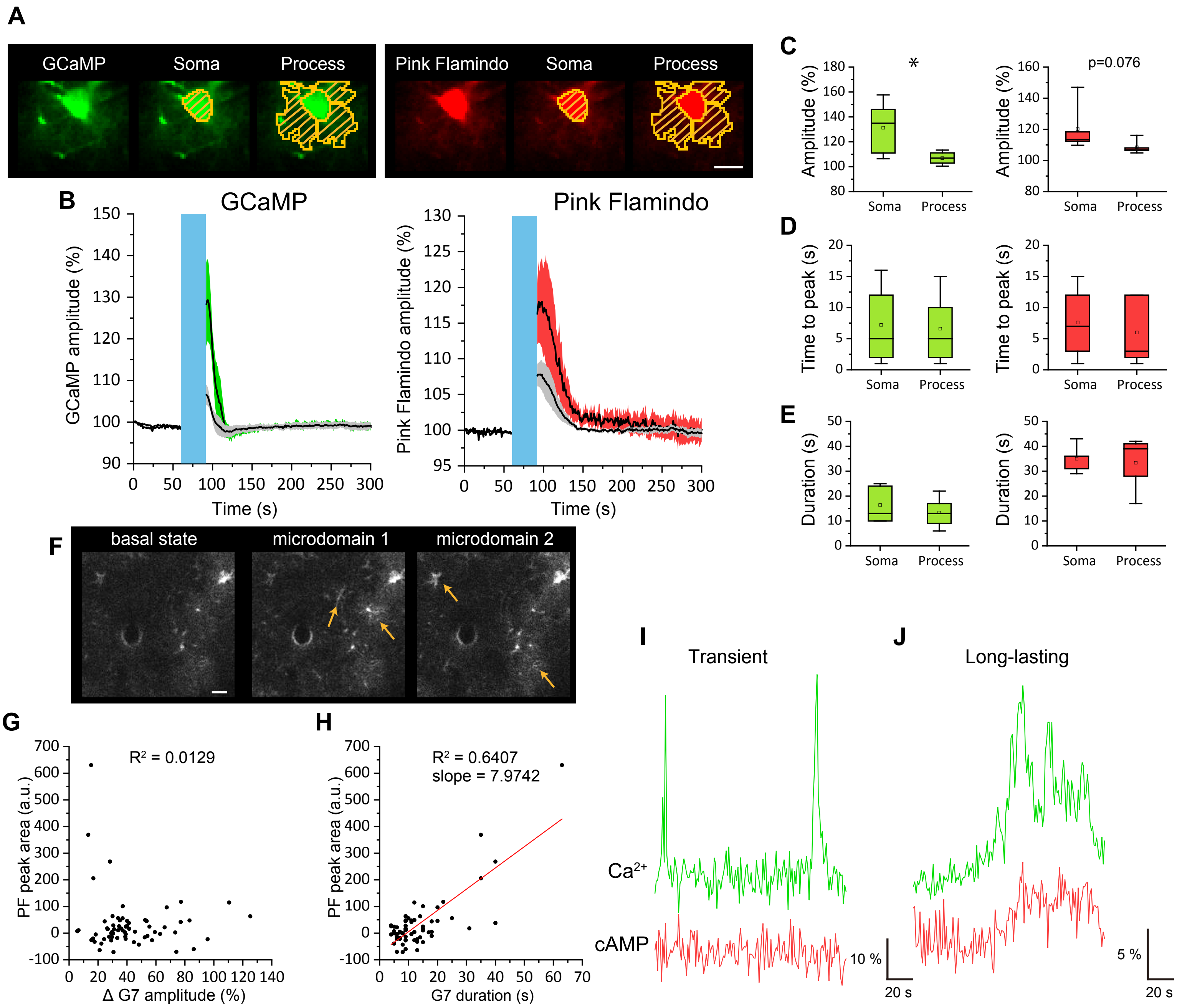

Supplementary Figure 12. Astrocytic processes exhibit cAMP elevation.

Simultaneous Ca^{2+} and cAMP imaging was performed and analyzed in astrocytic processes (A). Both Ca^{2+} and cAMP were elicited by 30-s PS with similar time courses as those from somas, but smaller peak amplitude (B, C, D, E). (n = 5 events from 5 mice, paired t-test, *p < 0.05). Black lines with colored shades represent cell bodies and black lines with gray shades represent processes.

Ca^{2+} activities in microdomain were obtained from awake mice. Orange arrows indicate microdomain Ca^{2+} activities (F). Most activities are transient type (I), whereas long-lasting microdomain Ca^{2+} activities appeared less frequently. The latter events accompanied mild cAMP elevation (J). PF peak area under curve and ΔG7 amplitude do not correlate (G). On the other hand, G7 duration is correlated with PF peak area (H). PF Peak area is calculated as the integral of PF signal for the period of ± 1s from the peak (n = 66 microdomains from 4 mice). Scale bars: 20 μm.

For all box plot figures, middle lines indicate the median, the squares are mean, the boxes show the inter quartile range between the 25th and 75th and upper and lower whisker indicate maximum and minimum values.

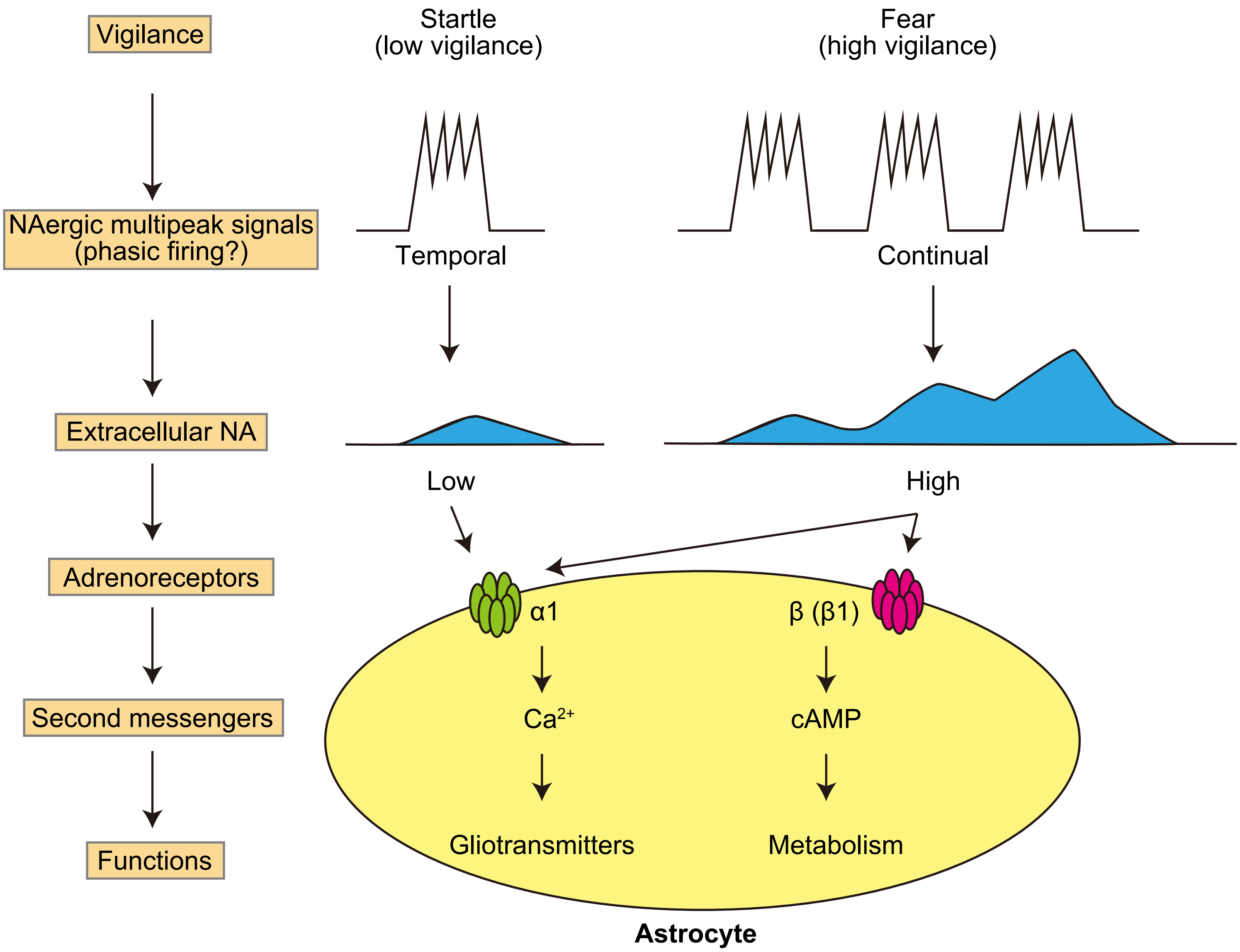

Supplementary Figure 13. Elevations of astrocytic second messengers depends on the level of vigilance.

Low vigilance states exemplified by face air puff startle induce temporal NAergic MP signals, which results in a low extracellular NA elevation. High-affinity α1-ARs are responsive to low extracellular NA and trigger astrocytic Ca^{2+} elevation. On the other hand, high vigilance states represented by fear induce continual NAergic MP signals, which elevate extracellular NA to a high level. Lo-affinity β-ARs can respond to the high extracellular NA level that causes astrocytic cAMP elevations in astrocytes.
