# Supplementary material for: Distinct temporal integration of noradrenaline signaling by astrocytic second messengers during vigilance
Source: Nat Commun. 2020 Jan 24;11:471. doi: 10.1038/s41467-020-14378-x (PMC6981284; doi:10.1038/s41467-020-14378-x)
Supplement: Supplementary file 4 — Description of Additional Supplementary Files [file 41467_2020_14378_MOESM4_ESM.pdf]

Description of Additional Supplementary Files

File Name: Supplementary Movie 1
Description: Ca^{2+} imaging by 5-s PS.

File Name: Supplementary Movie 2
Description: cAMP imaging by 30-s PS.

File Name: Supplementary Movie 3
Description: Extracellular NA imaging by 30-s PS.

File Name: Supplementary Movie 4
Description: Simultaneous LC/NA axons and astrocytic Ca^{2+} in MP signal.

File Name: Supplementary Movie 5
Description: Simultaneous LC/NA axons and astrocytic Ca^{2+} in SP signals.
